# Clinicopathological Profile of Thyroid Carcinoma in Young Patients: An Indonesian Single-Center Study

**DOI:** 10.1155/2022/9944083

**Published:** 2022-01-11

**Authors:** Agnes Stephanie Harahap, Desty Gusti Sari, Marini Stephanie, Alvita Dewi Siswoyo, Litta Septina Mahmelia Zaid, Diani Kartini, Maria Francisca Ham, Tri Juli Edi Tarigan

**Affiliations:** ^1^Department of Anatomical Pathology, Faculty of Medicine Universitas Indonesia, Dr. Cipto Mangunkusumo Hospital, Jakarta, Indonesia; ^2^Human Cancer Research Center, Indonesian Medical Education and Research Institute, Faculty of Medicine Universitas Indonesia, Jakarta, Indonesia; ^3^Faculty of Medicine Universitas Indonesia, Dr. Cipto Mangunkusumo Hospital, Jakarta, Indonesia; ^4^Department of Radiology, Faculty of Medicine Universitas Indonesia, Dr. Cipto Mangunkusumo Hospital, Jakarta, Indonesia; ^5^Department of Surgery, Faculty of Medicine Universitas Indonesia, Dr. Cipto Mangunkusumo Hospital, Jakarta, Indonesia; ^6^Department of Internal Medicine, Faculty of Medicine Universitas Indonesia, Dr. Cipto Mangunkusumo Hospital, Jakarta, Indonesia

## Abstract

**Introduction:**

Thyroid cancer is the third most common cancer that occurs in children and adolescents. Papillary thyroid carcinoma (PTC) is the most common type of thyroid malignancy. Although the mortality rate of thyroid malignancy in children is usually low, the disease recurrence is higher in children with more severe clinical presentation than in adults. This study aimed to determine the demographic and clinicopathological characteristics and outcome of pediatric and adolescent patients with thyroid malignancy in Indonesia.

**Methods:**

The retrospective study included all patients diagnosed with thyroid carcinoma aged <20 years, from January 1, 2015, to December 31, 2019. Twenty-nine subjects fulfilled the inclusion and exclusion criteria. We retrieved baseline characteristics, pathology features, TSH and fT4 status, radioactive iodine therapy data, and patients' outcomes. Then, data were analyzed using the chi-square or Fisher's exact method.

**Results:**

We identified 29 eligible subjects, including 3 boys and 26 girls. The most common type of thyroid carcinoma was PTC (96.5%), and follicular type (31%) was the predominant variant of PTC. Lymph node involvement occurred in 24% of patients, while distant metastasis occurred in 17.2% of patients with PTC. Twenty-four (82.7%) patients had stage 1 disease. Disease recurrence was recorded in 31% of patients during the study period with a median follow-up time of 24 months.

**Conclusion:**

PTC is the most frequent type of thyroid carcinoma among children and adolescents. This malignancy has a low mortality rate, but the recurrence rate remains high among younger patients than adults even during a short-term follow-up analysis. Distant metastasis and lymph node involvement are commonly found in this age group.

## 1. Introduction

Thyroid cancer is the third most common cancer that occurs in children and adolescents and is one of the most common endocrine malignancies in the pediatric population. [1] It has been reported that the incidence of malignant tumors in children and adolescents is around 0.5–3% and increases with age [2]. Papillary thyroid carcinoma (PTC) is the most common type of thyroid malignancy and contributes to about 70–80% of thyroid cancer cases in children [3, 4].

Age, radiation exposure of the head and neck region, family history, and environmental exposure are risk factors for thyroid malignancy [5, 6]. The mortality rate of thyroid malignancy in children is usually <10%, but the recurrence rate is higher in children who are diagnosed when younger than 15 years with clinical presentation usually more severe than in adults [2, 7].

Thyroid malignancy in younger patients has different characteristics than in adults in terms of clinical presentation, pathogenesis, and outcome owing to its biologic and molecular differences [7]. Young patients with thyroid malignancy commonly do not show signs and symptoms but are diagnosed with malignant disease rather suddenly; therefore, it is often known as the silent killer [6].

Thus far, only a few studies have been conducted on thyroid malignancy in children compared with adults, especially in Indonesia. This study aimed to determine the demographic and clinicopathological characteristics and outcome of pediatric and adolescent thyroid malignancy, especially PTC. A deeper understanding of PTC in this population is required to further improve the outcome of pediatric patients.

## 2. Methods

This retrospective study was conducted by collecting data from the archives of the Department of Anatomical Pathology in our institution. Data were collected using a total sampling technique. The inclusion criteria in this study were patients diagnosed with thyroid carcinoma confirmed with examination after surgical intervention, age <20 years old from January 1, 2015, until December 31, 2019, in Dr. Cipto Mangunkusumo Hospital. An inadequate or light paraffin block was excluded in this study. We also obtained data from the medical record unit to complete the clinical data collection.

We collected the clinical data and laboratory thyroid parameters (TSH and fT4 status) which were obtained through medical records. The thyroid function parameters were recorded prior to surgery. Fine needle aspiration cytology (FNAC) diagnosis was made by using The Bethesda Scoring System [8]. The size of tumor nodules was assessed by direct measurement at macroscopic pathology examination. All histopathology variables such as tumor types, variant, thyroid inflammation background, lymphovascular invasion (LVI), capsular invasion, soft tissue invasion, and surgical margin were assessed using hematoxylin and eosin (H&E) staining and reviewed by two pathologists. Tumor types and variants were determined based on tumor classification by The World Health Organization (WHO). Tumor staging was performed using the Tumor, Node, Metastasis (TNM) classification system (7^th^ edition) for patients younger than 45 years, which classified them into two groups: stage 1 (any T, any N, M0) and stage 2 (any T, any N, M1) [9]. The MACIS (Metastases, Age, Completeness of Resection, Invasion, Size) score was used as a prognostic factor for pediatric patients with PTC and was calculated as 3.1 + 0.3 × tumor size (cm) + 1 (if incompletely resected) + 1 (if locally invasive) + 3 (if distant metastasis present) [10, 11]. Then, we collected the radioactive iodine therapy's (RAI) data of patients. Disease recurrences were assessed as outcome. Disease recurrence was assessed if the same malignancy appeared after the previous surgical intervention during the studies period.

All data were analyzed using IBM SPSS Statistics version 25 (IBM Corp, NY). The numerical data are presented as mean ± standard deviation if the data are normally distributed, or as median and range if the data are not normally distributed. Categorical data are presented as numbers and percentages. Hypothesis testing was done by comparing the pathological characteristics with the recurrence data. These data were analyzed using chi-square or Fisher's exact test where appropriate. For all analyses, *p* < 0.05 was considered to indicate statistical significance. This retrospective study was approved by the local ethics committee and institutional review board of our institution. Informed consent was waived by the institutional review board because this study was using archive data.

## 3. Results

### 3.1. Clinical and Biochemical Data

We identified 29 eligible subjects including 3 (10.3%) boys and 26 (89.7%) girls. The mean age at initial diagnosis of thyroid carcinoma was 14 ± 3.7 years. The youngest patient was diagnosed at 5 years old.

The thyroid hormone function (TSH and fT4 levels) was measured in 23 (79.3%) patients. Meanwhile, TSH and fT4 data were not available in six patients' medical records. The median TSH level among subjects was 1.37 nIU/mL (range: 0.0025–83.50), and the median fT4 level was 1.08 (range: 0.49–4.25). Most patients (44.8%) had low TSH levels and 31% had low fT4 levels. That level was below the baseline reference of our hospital.

### 3.2. FNAC Bethesda Scoring

Twenty (68.96%) of the 29 patients with thyroid carcinoma underwent FNAC examination; of these, one patient had Bethesda I scoring, five showed Bethesda II, three showed Bethesda III, three showed Bethesda IV, five showed Bethesda V, and three showed Bethesda VI. Six patients showed false-negative results (one Bethesda I and 5 Bethesda II), possibly owing to inaccurate sampling (Table 1). Nine patients did not undergo FNAC examination because some pediatric patients were uncooperative; in general, FNAC was performed without local anesthesia. So, the patient was immediately operated on without having an FNAC examination.

### 3.3. Pathological Findings

**Figure 1:**
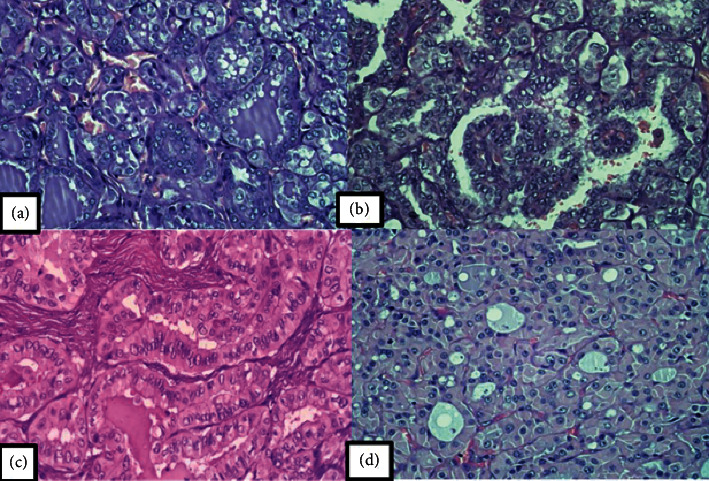
(a). A case of PTC showing follicular structures (H&E staining: 400x). (b). The tumor cells were overlapped and showed typical ground glass nuclei (H&E staining: 400x). (c). A case of tall cells variant of PTC, the tumor cells showed a height of 2–3 times the width and abundant eosinophilic cytoplasm (H&E staining: 400x). (d). A case of Hurthle cell carcinoma (H&E staining: 400x).

The most common type of thyroid carcinoma was PTC (96.5%), with the follicular type (31%) being the predominant variant of this malignancy, followed by the tall cell (27.6), classic (20.7%), and microcarcinoma (17.2%) types. One patient had Hurthle cell carcinoma with minimal invasion (Figure 1). Fifteen cases (51.7%) showed multifocal tumor. The mean tumor size was 2.5 ± 2.6 cm (range: 0.3–15 cm). Microscopic examination showed that seven (24.1%) patients had lymphovascular invasion (LVI) and three (10.3%) had perithyroidal soft tissue invasion. From the resection margin or surgical margin, 17.2% of tumors were identified microscopically at the margin site, while 75.9% of tumors had a free surgical margin. The surgical margin of the other two (6.9%) cases could not be assessed given the lack of margin stain. The baseline characteristics of the study subjects are summarized in Table 2.

Only 24.1% of patients with PTC underwent lymphadenectomy, and all of them showed lymph node involvement. There were 2 patients with central lymph node dissections, 2 patients with lateral lymph node dissections, and 1 patient with central and lateral lymph node dissection, and 2 cases were unknown. The tall cell variant of PTC was the most common variant that showed lymph node involvement. Five (71.4%) patients with LVI had lymph node involvements (*p*=0.003).

Five (17.2%) of 29 patients experienced distant metastasis, with all five having lung metastases. Four patients had metastases with a mean of 6 months after PCT diagnosis and 1 patient had pulmonary metastases at a first hospital visit. One patient had lung metastases followed by metastasis of the mediastinum, cranial/parietooccipital bone, pelvic bone, and left cruris bone. Another patient had lung metastases followed by metastasis of the mediastinum and mandibular. The tall cell variant was the most common variant of PTC with distant metastasis. However, no patient with the microcarcinoma variant had distant metastases. Out of five cases with distant metastasis, four showed capsular invasion (*p*=0.013) and LVI (*p*=0.007).

### 3.4. Tumor Staging

Twenty-four (82.7%) patients had stage 1 disease and five (17.2%) patients had stage 2 disease. The TNM staging details of all patients are summarized in Table 3.

### 3.5. MACIS Score

By using Bethesda score 4 as the cut-off [10, 11], 28 patients with PTC showed mean MACIS score of 4.8 (range: 3.25–8.75). All pathological characteristics were compared and there were no statistically significant differences between the MACIS scores with pathological characteristics. However, we found that most PTCs with the tall cell variant showed higher MACIS scores than the other variants.

### 3.6. Treatment

#### 3.6.1. Surgery

All patients in this study underwent surgical intervention and 7 of them also underwent cervical lymphadenectomy. Total thyroidectomy was done in 15 (51.7%) patients with PTC. Hemithyroidectomy was done in nine (31%) patients, and five (17.2%) patients underwent subtotal thyroidectomy. More than half the patients with total thyroidectomy had distant metastases (60%) and experienced recurrence (55.6%) during follow-up.

#### 3.6.2. Radioactive Iodine (RAI)

Out of 29 patients, only 11 (37.9%) underwent RAI therapy 4–5 weeks after surgical intervention in patients with total thyroidectomy. Some patients were lost to follow-up. And other patients refused to use RAI because of complications possibility of RAI to the gonads. The age range of patients who received RAI therapy was 10–20 years (mean: 15.09 years and median: 15 years). Of 11 patients who received RAI, seven had lymph node metastases, five had lung metastases, and two had other distant metastases. In some cases, these patients may have had 1–2 organ metastasis at the same time. Cumulative dose of I-131 varied from 30 mCi (*n* = 4), 60 mCi (*n* = 1), 100 mCi (*n* = 5), and 150 mCi (*n* = 1) with a mean cumulative I-131 dose of 75 ± 41 mCi and a median I-131 dose of 100 mCi. The indication and fixed dose of I-131 was determined based on clinical consideration of patient's disease stage and body weight.

The RAI therapy details of all patients are summarized in Tables 1 and 4.

### 3.7. Outcome

All cases of recurrence occurred in girls, with the most common variant of PTC being the tall cell (44.4%) and classic (44.4%) types. Follicular variant of PTC showed no recurrence (*p*=0.027). Out of seven cases with LVI, only one showed recurrence. This patient also showed soft tissue and capsular invasion. Surprisingly, only 1/5 cases with positive margin and 6 cases with negative margin showed recurrence. In this study, tumor recurrence was not correlated with tumor size (*p*=0.694) and lymph node metastases (*p*=0.642); further, there were no distant metastases noted during the follow-up. Five (55.6%) patients with tumor recurrence previously underwent total thyroidectomy, 3 (33.3%) patients underwent subtotal thyroidectomy, and only 1 (11.1%) underwent hemithyroidectomy (Table 5).

The correlation between pathological characteristics with recurrence, lymph node involvements, and distant metastasis are summarized in Table 6.

## 4. Discussion

Thyroid carcinoma usually affects more girls than boys [12]. Hay et al. [13] showed that the average age of patients with PTC was 14 years (range: 3–18 years), with 75% of their study subjects being girls. In this study, girls in the age range of 5–20 years more commonly had thyroid carcinoma than boys in the same age range, with the mean age being 14 ± 3.7 years. Some people suggest that the female sex dominates PTC cases which reflects the natural incidence of this malignant tumor [14].

The role of TSH in increasing the risk of PTC is still debatable. The pathogenesis of PTC is thought to be influenced by a high TSH level in mouse studies [15]. In a recent study [16], the authors found that low TSH levels can increase the risk of PTC in female patients. In contrast, a high TSH level was found to be correlated with the risk of PTC in male patients. The median value of TSH level in our study was low, with most subjects being girls. This finding was similar to that reported in a previous study. The underlying mechanism is still unclear, but genetics is suggested to play a role in these cases [17, 18].

The vast majority of thyroid cancers in this study are PTC (96.5%), which is consistent with other published studies [12, 19]. Only one patient was diagnosed with Hurthle cell carcinoma with minimal invasion. This case showed the presence of a single nodule measuring 4 cm with capsular and vascular invasion, but the recurrence and metastasis status were unknown owing to loss of follow-up. More aggressive thyroid carcinomas such as poorly differentiated ones or anaplastic carcinomas were not found.

Among the patients diagnosed with PTC, the follicular variant was the most common, followed by the tall cell variant. The classic variant was found in 20.7% of patients, while, in another large series study by Balachandar et al. [20], the classic variant was the predominant type (48%), and the tall cell variant was found only in 12.9% of patients. Out of these eight patients with the tall cell variant, four showed recurrence and three had distant metastasis. These findings are consistent with the natural aggressive behavior of the tall cell variant. Five (17.2%) patients with PTC had the microcarcinoma variant. This variant usually shows good prognosis and less aggressive behavior [21]. Out of the five patients, only one patient had recurrence and the most plausible explanation for this aggressive behavior was tumor multifocality.

Unlike adults, thyroid cancer in children usually presents with severe disease at the time of diagnosis. Most pediatric patients with differentiated thyroid carcinoma (DTC) usually experience regional nodal involvement, seen in 60–80% of the population; meanwhile, in adults, regional nodal involvement occurs in 30–40% of cases [22]. Seven patients with PTC in our study had lymph node involvements. The presence of LVI was statistically significant with lymph node involvement in our population.

Distant metastasis is usually found in children with PTC. Eight of 42 patients with PTC in another published study experienced distant metastases [23]. Among them, 88% had lung metastasis and 12% of patients had bone metastasis [23]. Cervical node or distant metastases, especially lung metastases, is more often found in children with DTC than in adults [24]. Distant metastasis was found in 17.2% of subjects in their study. All of them had lung metastases. Distant metastases were commonly found in the tall cell variant. This variant shows mitotic activity and vascular invasion more often and is understood to be a more aggressive variant than others [25]. Out of five cases with distant metastasis, four had capsular invasion (*p*=0.013) and four had LVI (*p*=0.007).

Among the 20 patients who underwent FNAC, 6 (30%) showed false-negative results likely because of inaccurate sampling. Of these patients with false-negative results, two underwent FNAC examination without USG guidance. There was no information about the USG guidance in the remaining four cases. The six false-negative cases had a tumor nodule size ranging from 0.5 to 3 cm. Three patients with atypia of undetermined significance (AUS) had the tumor size ranging from 0.3 to 15 cm. Among them, two patients underwent FNAC without USG guidance. According to the Bethesda system, AUS has a 10–30% risk of malignancy, although AUS can also imply that the sample is inadequate. Kizilgul et al. [26] reported that there was no significant difference in the false-negative rate in nodule sizes with a cut-off of 4 cm. In contrast, another study [27] reported the opposite results, with a higher false-negative rate in nodules >4 cm. Meanwhile, Kuru et al. [28] reported four false-negative cases in nodules >4 cm. Two patients had microcarcinoma and one had a follicular variant, which was similar to our report.

The AJCC TNM classification is widely used in the adult population to describe the extent of disease and disease prognosis. However, there is no validated staging system for thyroid carcinoma in children, and the other adult staging systems are not suitable for pediatric cases due to different clinical settings [29]. Therefore, several other staging systems such as AGES, AMES, MACIS, and ATA have been proposed to achieve better accuracy in pediatric prognosis than the AJCC TNM classification [6]. Powers et al. [10] and Jang et al. [11] concluded that the MACIS score was effective to predict tumor aggressiveness and recurrences of PTC in children and adolescents. MACIS score >4.0 is significantly correlated with more aggressive tumors and a higher risk of recurrent or persistent disease in PTC than patients with MACIS score <4.0. Meanwhile, there were no statistically significant differences between a cut-off value of 4.0 in the MACIS score and all pathological characteristics in our study. However, we found that most PTC cases with the tall cell variant showed higher MACIS scores than the other variants.

Based on the American Thyroid Association, [30] the choice of surgical intervention is based on tumor size, extrathyroidal extension, and lymph node metastases or distant metastases. The near-total or total thyroidectomy is performed in patients with large tumor size (clinical T4), and the evidence of lymph node (clinical N1) or distant metastases (clinical M1). The use of adjuvant RAI after surgical intervention in pediatric PTC patients without lymph nodes or distant metastases is still controversial. The ATA guidelines recommend RAI therapy for iodine-avid residual nodal/locoregional disease not amenable to surgery and/or distant metastatic disease [24]. TSH-stimulated Tg can also guide management. RAI therapy should be considered in patients with Tg > 2 ng/mL. Of our 29 patients, only 11 underwent RAI based on disease stage (residual cervical disease, locoregional, and metastases). In this study, the cumulative doses of RAI ranged from 30 mCi to 150 mCi.

The recurrence of PTC in children and adolescents occurs in 15% and 40% of the population and is usually found 20 years until 30 years after the initial diagnosis. [31] In our study, 9 (31%) patients had tumor recurrence in 1–4 years after the initial diagnosis. Tall cell and classic types were the predominant variants to experience tumor recurrence. A recent study [32], showed that not only younger age at diagnosis but also recurrence rate, tumor multifocality, and tumor size were important predictors of poor prognosis and recurrence in PTC in the pediatric population. Tall cell is an aggressive variant of PTC and is associated with a high recurrence rate and, hence, the treatment must also be more aggressive.

Despite this, the prognosis of DTC in children is excellent. The 10-year mortality is less than 10% with 98% overall survival [22] in our institution.

This retrospective study has some limitations. Not all patients were tested for thyroid function prior to diagnosis, and the sample size of the study was relatively small. Besides, this study also had a short follow-up time. We hope other researchers can use our results for a basic study of thyroid carcinoma, especially in children and adolescents. A prospective study can better identify and evaluate the prognosis of this malignancy, the recurrence rate, and associated mortality in the next 10 years and until 20 years after diagnosis and treatment.

## 5. Conclusion

We found that PTC is the most common type of thyroid carcinoma among children and adolescents. Follicular, classic, and tall cell types were the most commonly found PTC variants in this study, and the tall cell variant was recognized for its aggressive behavior. This malignancy had a low mortality rate, but the recurrence rate was high among young patients even in the short duration of follow-up. The tall cell variant has a high recurrence rate and, hence, the treatment of this variant must also be more aggressive. Distant metastasis and lymph node involvement are commonly found in this population.

Malignancy staging is crucial to managing and evaluating the disease in children. The use of adjuvant RAI after surgical intervention in pediatric PTC patients without lymph nodes or distant metastases is still debatable.

## Figures and Tables

**Table 1:** Baseline characteristics of study subjects.

Characteristic	Number of patients (*n* = 29)

Age, years (mean ± SD, range)	14 ± 3.7, 5–20
Sex (*n*, %)	
Boys	3 (10.3)
Girls	26 (89.7)
Thyroid parameters (median, range)	
TSH (*μ*IU/mL)	1.37, 0.0025–83.50
fT4 (ng/dL)	1.08, 0.49–4.25
Pathological characteristics	
Tumor size, cm (mean ± SD), (range)	2.5 ± 2.6, 0.3–15
Tumor type	
Papillary thyroid carcinoma (PTC)	28 (96.5)
Hurthle cell carcinoma	1 (3.4)
PTC variant (*n*, %)	
Classic	6 (20.7)
Tall cell	8 (27.6)
Follicular	9 (31)
Microcarcinoma	5 (17.2)
Multifocality	15 (51.7)
Background thyroid inflammation	0 (0)
Lymphovascular invasion (LVI)	7 (24.1)
Soft tissue invasion	3 (10.3)
Capsular invasion	8 (27.6)
Surgical margin	
Positive margin	5 (17.2)
Negative margin	22 (75.9)
Unknown	2 (6.9)
Distant metastasis	5 (17.2)
Intervention (*n*, %)	
Surgical intervention	
Hemithyroidectomy	9 (31.0)
Subtotal thyroidectomy	5 (17.2)
Total thyroidectomy	15 (51.7)
Radioactive iodine (RAI) therapy	11 (37.9)
MACIS score (mean, range)	4.8, 3.25–8.75
Recurrence	9 (31)
Mortality	0 (0)

SD: standard deviation; *n*: number; MACIS: distant metastasis, patient age, completeness of resection, local invasion, and tumor size; *μ*IU: microinternational unit; mL: milliliter; ng: nanogram; dL: deciliter.

**Table 2:** FNAC Bethesda scoring details.

FNAC Bethesda scoring	Papillary thyroid carcinoma				Hurtle cell carcinoma (*n* = 1)	Number of patients (*n* = 29)
	Classic (*n* = 6)	Tall cells (*n* = 8)	Follicular (*n* = 9)	Microcarcinoma (*n* = 5)		

(I) Nondiagnostic or unsatisfactory	0	1	0	0	0	1
(II) Benign	2	0	0	3	0	5
(III) Atypia of undetermined significance or follicular lesion of undetermined significance	0	0	3	0	0	3
(IV) Follicular neoplasm or suspicious for a follicular neoplasm	0	1	2	0	0	3
(V) Suspicious for malignancy	1	1	1	1	1	5
(VI) Malignancy	0	2	1	0	0	3
Not available	3	3	2	1	0	9

n: number.

**Table 3:** TNM staging details of patients.

TNM staging			Number of patients (%)	Radioactive iodine therapy doses			
T	N	M		30 mCI (*n* = 4)	60 mCI (*n* = 1)	100 mCI (*n* = 5)	150 mCI (*n* = 1)

T1a	N0	M0	5 (17.2)	0	0	0	0
T1b	N0	M0	10 (34.5)	2	1	1	0
	N0	M1	2 (6.9)	1	0	0	1
	Nx	M0	1 (3.4)	0	0	1	0
	N1	M1	1 (3.4)	0	0	0	0

T2	N0	M0	3 (10.3)	1	0	0	0
	N0	M1	1 (3.4)	0	0	0	0
	N1	M0	2 (6.9)	0	0	1	0
	NX	M0	1 (3.4)	0	0	1	0
	NX	Mx	1 (3.4)	0	0	1	0

T3	N0	M0	1 (3.4)	0	0	0	0
	N1	M1	1 (3.4)	0	0	0	0

T: tumor; N: node; M; metastasis; mCI: millicuries; *n*: number.

**Table 4:** Surgical interventions with the outcome of PTC.

Surgical interventions	LN involvements (*n* = 7)	Distant metastasis (*n* = 5)	Recurrences (*n* = 9)	Total (*n* = 29)

Hemithyroidectomy (*n*,%)	0 (0)	0 (0)	1 (11.1)	9 (31)
Subtotal thyroidectomy (*n*,%)	2 (28.6)	2 (40)	3 (33.3)	5 (17.2)
Total thyroidectomy (*n*,%)	5 (71.4)	3 (60)	5 (55.6)	15 (51.7)

LN: lymph node; LVI: lymphovascular invasion; *n*: number.

**Table 5:** Radioactive iodine therapy's doses.

Doses	Staging		Number of patients (*n* = 29)	Residual thyroid tissue (*n* = 14)	Metastasis location		
	Stage I (*n* = 24)	Stage II (*n* = 5)			Lymph node metastasis (*n* = 7)	Lung metastasis (*n* = 5)	Other distant metastases (*n* = 2)

I-131 30 mCI	3	1	4	4	1	1	1
I-131 60 mCI	1	0	1	1	0	0	0
I-131 100 mCI	4	1	5	4	2	1	1
I-131 150 mCI	0	1	1	1	1	1	0
Not available	16	2	18	4	3	2	0

Based on whole body scan I-131 or ultrasonography. mCI: millicuries; I-131: iodine-131; *n*: number.

**Table 6:** The correlation between pathological characteristics with recurrence.

	Recurrences (*n* = 9)	*p* value	Total (*n* = 29)

Sex (*n*, %)			
Boys	(0)	0.530	3 (10.3)
Girls	9 (100)		26 (89.7)
PTC variant (*n*, %)			
Classic	4 (44.4)	0.056	6 (20.7)
Tall cell	4 (44.4)	0.209	8 (27.6)
Follicular	0 (0)	0.027	9 (31)
Microcarcinoma	1 (11.1)	1.000	5 (17.2)
Tumor size (*n*, %)			
<2 cm	3 (33.3)	0.694	12 (41.4)
≥2 cm	6 (66.7)		17 (58.6)
Multifocality (*n*, %)	4 (44.4)	0.689	15 (51.7)
LVI (*n*, %)	1 (11.1)	0.382	7 (24.1)
Soft tissue invasion (*n*, %)	1 (11.1)	1.000	3 (10.3)
Capsular invasion (*n*, %)	1 (11.1)	0.371	8 (27.6)
Surgical margin (*n*, %)			
Positive margin	1 (11.1)	0.110	5 (17.2)
Negative margin	6 (66.7)		22 (75.9)
Unknown	2 (22.2)		2 (6.9)
LN involvements (*n*, %)	3 (33.3)	0.642	7/7 (100)

LN: lymph node; LVI: lymphovascular invasion; *n*: number.

## Data Availability

The data used to support the findings of this study are available from the corresponding author upon request.
